# Biomarkers for predicting the efficacy of immune checkpoint inhibitors

**DOI:** 10.7150/jca.65012

**Published:** 2022-01-01

**Authors:** Chengji Wang, He-nan Wang, Liang Wang

**Affiliations:** 1 Beijing Tongren Hospital, Capital Medical University, Beijing 100730, China.; 2 Department of Hematology, Beijing Tongren Hospital, Capital Medical University, Beijing 100730, China.; 3 Beijing Advanced Innovation Center for Big Data-Based Precision Medicine, Beihang University & Capital Medical University, Beijing Tongren Hospital, Beijing 100730, China.

**Keywords:** Predictive biomarkers, PD-L1 expression, circulation biomarkers, immunotherapy, hyper progressive disease

## Abstract

Immune checkpoint blockade has vastly changed the landscape of cancer treatment and showed a promising prognosis for cancer patients. However, there is still a large portion of patients who have no response to this therapy. Therefore, it's essential to investigate biomarkers to predict the efficacy of immune checkpoint inhibitors. This article summarizes the predictive value of established biomarkers, including programmed cell death ligand 1(PD-L1) expression level, tumor mutational burden, tumor-infiltrating lymphocytes, and mismatch repair deficiency. It also addresses the predictive value of tumorous mutations, circulation factors, immune-related factors, and gut microbiome with immunotherapy treatment. Furthermore, some of the emerging novel biomarkers, and potential markers for hyper progressive disease are discussed, which should be validated in clinical trials in the future.

## Introduction

Programmed cell death protein 1/programmed cell death ligand 1 (PD-1/PD-L1) is believed to be a pathway closely related to tumor growth and immune evasion 1. PD-1/PD-L1 axis is considered to inhibit proliferation and activation of T cells, breaking the balance of immune surveillance and immune resistance 2. PD-1 and PD-L1 blockade has greatly improved the prognosis of cancer patients and favorable outcomes have been observed since Food and Drug Administration (FDA) approved the first immune checkpoint inhibitor (pembrolizumab) in 2014. Some biomarkers are believed to have power in predicting the effect of PD-1/PD-L1 antibodies, such as PD-L1 expression levels and tumor mutation burden (TMB) 3,4.

However, not all patients could obtain clinical benefits from anti-PD-1/PD-L1 therapy, and still a large number of patients, with shorter progression-free survival (PFS), have no response to PD-1 inhibitors, which limited the clinical applications of anti-PD-1/PD-L1 therapy 5. Predictive biomarkers can be used to select patients most likely to be benefited from immunotherapy, reducing costs and treatment-related adverse reactions. This article reviewed current potential biomarkers by categories of tumor-related factors, immune-related factors, and factors detected in tumor tissue and peripheral blood. The effect of biomarkers for hyper progressive disease (HPD) was also discussed, but the role of these biomarkers is still controversial with the current available clinical data.

## Established biomarkers

### PD-L1 expression

PD-L1 expression has remained to be the focus of various studies since the early stage of immunotherapy. Based on the results of the phase III KEYNOTE-024 trial, patients treated with pembrolizumab showed much longer progression-free survival (PFS) and overall survival (OS) than those treated with platinum-based chemotherapy, and patients of both groups had at least 50% PD-L1 expression on their tumor cells 3. In the KEYNOTE-052 trial, a phase II study of pembrolizumab for patients with urothelial carcinoma, the objective response rate (ORR) in the group with PD-L1 expression higher than 10% was significantly better than that in the group with PD-L1 expression lower than 1% (39% vs 11%) 6.

The efficacy of immune checkpoint inhibitors (ICIs) varied with tumor types, but it is generally believed that high PD-L1 expression level is associated with favorable clinical outcomes to anti PD-1/anti PD-L1 therapy (Table 1). Schmid et al. 7 observed that PD-L1 expression on tumor-infiltrating immune cells as a percentage of tumor area is predictive in advanced triple-negative breast cancer patients treated with atezolizumab and nab-paclitaxel. Patients in the PD-L1 positive group (> 1%) had a longer median overall survival (25 months vs 21.3 months) than all patients 7. But in the OAK study of non-small cell lung cancer (NSCLC) patients treated with atezolizumab, patients with low or negative PD-L1 (PD-L1 expression < 1%) also had a longer median OS than those treated with docetaxel (12.6 months vs 8.9 months), while clinical benefit was more significant in patients with positive PD-L1 expression 8. This finding suggests PD-L1 expression level is not the predictive determinant for efficacy of ICIs.

Recently, PD-L1 structural rearrangements (PD-L1^MUT^) was reported as a potential biomarker for response to immunotherapy in patients with relapse/refractory natural-killer/T cell lymphoma. This frequent somatic mutation led to the disruption of the 3'-UTR of PD-L1 gene, was found to be significantly enriched in the responders to pembrolizumab (4 of 9) compared to the non-responders (0 of 10) 9.

Besides PD-L1 expression levels on T cells and tumor cells, Liu et al. 10 found that high-level of PD-L1 expression on CD68^+^ macrophages correlated with longer OS in NSCLC patients treated with immunotherapy. PD-L1 levels on macrophages were significantly associated with PD-L1 expression on tumor cells and CD8^+^ T-cell infiltration 10. However, defective and controversial aspects about the predictive role of PD-L1 could not be ignored. Not all predictions by PD-L1 expression for immunotherapy were positively correlated. Motzer et al. 11 reported that PD-L1 negative group (<1%) showed a longer median PFS (27.4 months vs 21.8 months) than PD-L1 (>1%) group in advanced clear-cell renal-cell carcinoma patients treated with nivolumab. Based on the results of CheckMate 040, a study of advanced hepatocellular carcinoma treated with nivolumab, PD-L1 predictive effect was not that significant: objective response in PD-L1 positive (cut-off value was 1%) group was 26%(9/34), while in PD-L1 negative group, that was 19%(26/140) 12. Remarkable intra-tumoral heterogeneity of PD-L1 expression was observed across breast cancer sub-types and stages 13. Ilie et al. 14 discovered that PD-L1 expression was often inconsistent between removed tissues and matched biopsy specimens (the overall discordance rate was 48%), which was measured by highly sensitive SP142 immunohistochemical (IHC) assay. Hong et al. 15 found that the heterogeneity of PD-L1 expression was influenced by anatomic locations of distant metastases and treatment stages in NSCLC patients. The course of immunotherapy also changed PD-L1 levels 15. These factors would result in inaccurate PD-L1 expression measurements from biopsies or surgically removed specimens.

IHC is widely used to assess the level of PD-L1 expression, which includes 4 main IHC assays: 22C3, 28-8, SP142, and SP263. Hirsch et al. 16 demonstrated that 28-8, 22C3 and SP263 were highly consistent in evaluating the PD-L1 expression on tumor cells, while the positive rate of SP142 antibody was lower compared with the other three assays. The discordance of PD-L1 expression with two rabbit monoclonal antibodies, E1L3N and SP142, was also observed in NSCLC patients 17. And no consensus of the cut-off value of PD-L1 expression had been reached across various tumor types.

Besides IHC, novel measurement of PD-L1 expression is being investigated. Conroy et al. 18 demonstrated that the PD-L1 mRNA expression, which was measured by next generation RNA sequencing, was associated with PD-L1 expression levels in a study of 209 patients. Furthermore, non-invasive positron emission tomography (PET) for evaluating PD-L1 expression levels was proved to be valid in immunotherapy effect prediction 19,20. Bensch et al. 20 claimed PET for PD-L1 status evaluation was more relevant to clinical response than IHC or RNA sequencing for PD-L1 levels. However, challenges like how to interpret and integrate PET data into medical practice are remained to be solved 21.

Although detection of PD-L1 expression levels is the major way to predict the efficacy, its predictive value might get improved when combined with other biomarkers 22, which would be further explained in this article.

### Tumor infiltrating lymphocytes (TILs)

TILs are crucial components of tumor microenvironment, which is related to tumor growth and regression. Tumeh et al. 23 found that proliferation of intra-tumoral CD8^+^ T cells was associated with tumor regression in metastatic melanoma patients treated with pembrolizumab. Furthermore, more T-cell antigen receptors (TCRs) and PD-1^+^ CD8^+^ T cells were observed inside and on the edge of the invasive tumors of the responders. Thommen et al. 24 reported that high baseline levels of PD-1^+^ CD8^+^ T cells (>1%, assessed by multiple immunohistochemistry) was correlated with longer OS in advanced NSCLC patients treated with nivolumab. CD45RO^+^ T cells were reported as independent predictive factors for favorable outcomes of immunotherapy across various cancers, while FOXP3^+^ Treg cells were considered to be related to poor prognosis 25-27.

**Figure 1:**
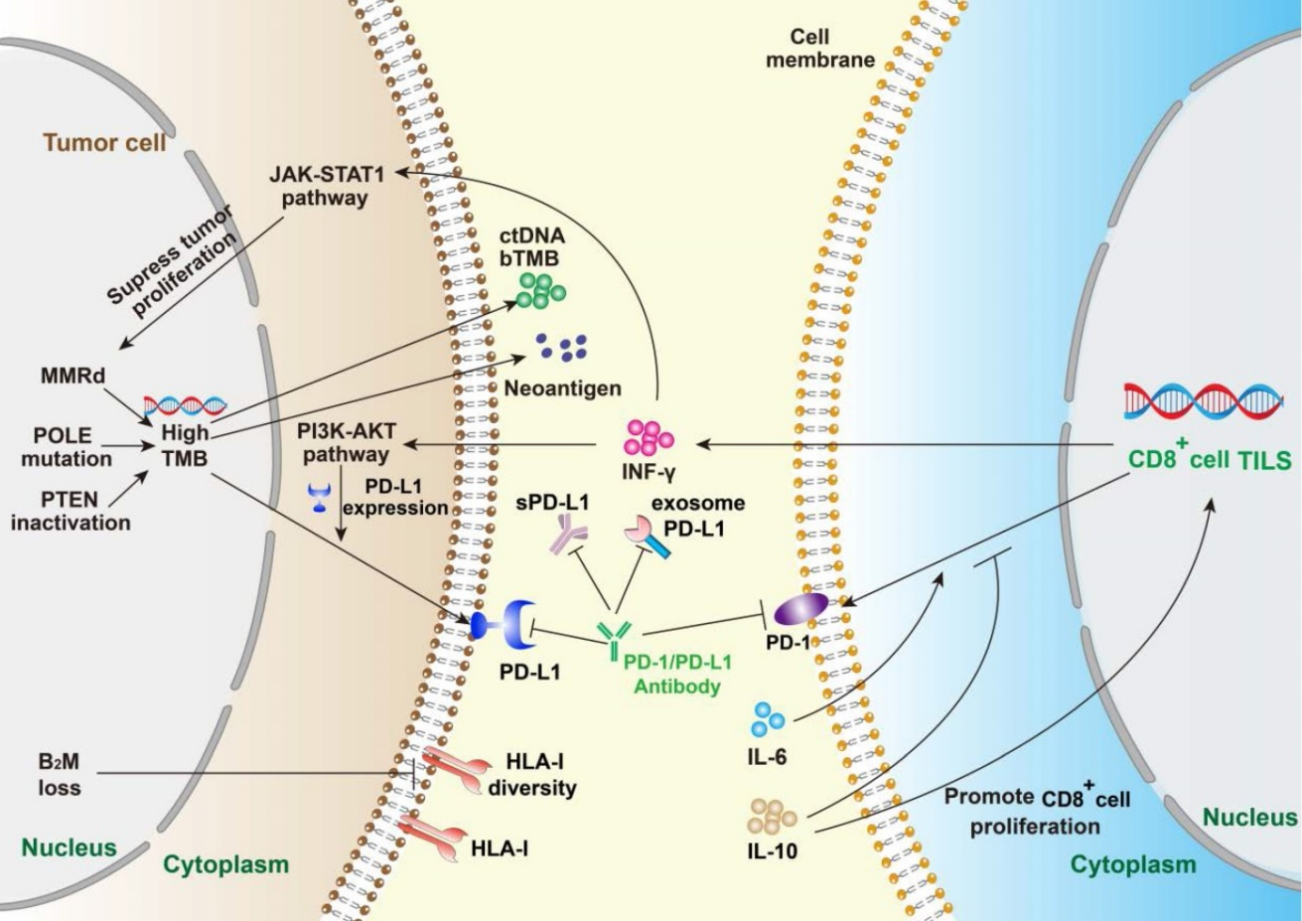
** Summary of mechanism of PD-1/PD-L1 and anti PD-1/PD-L1 immunotherapy.** The efficacy of PD-1/PD-L1 antibodies therapy is mainly predicted by PD-L1 expression, tumor infiltrating lymphocytes, tumor mutational burden and peripheral cytokines. PD-L1 expression reflects immune resistance, which is the target of PD-1/PD-L1 inhibitors. Mismatch repair deficiency (MMRd) and mutations of related genes will contribute to high TMB. Meanwhile, TMB enhances immunogenicity, which can be detected in the periphery. B_2_M loss suppresses the expression of HLA-I and leads to ICIs escape. IFN-γ, mainly derived from TILs, can enhance immune activity and inhibit tumor proliferation, but can also up-regulate the expression level of PD-L1 on tumor cells. IL-6 and IL-10 mainly play a regulatory role by affecting the PD-1 expression on immune cells.

Chen et al. 28 classified tumor microenvironment into four immune types by TILs (presence/absence) and PD-L1 expression (positive/negative) to evaluate clinical benefits of immunotherapy (Table 2). The cut-off values of TILs and PD-L1 expression were adjusted for various tumor types (Figure 1).

In metastatic melanoma and NSCLC patients, TILs and high PD-L1 expression levels were observed to be correlated with significantly longer OS and better prognosis 29,30. But resistance to immunotherapy in tumor microenvironment immune type 1(TMIT1) patients with urothelial carcinoma was also observed 31. Kim et al. 32 summarized and concluded that about 45% to 50% of patients had primary resistance to immunotherapy, but this data was influenced by the detection methods of PD-L1 expression and TILs, for example, the initial sampling of PD-L1 expression evaluation, the effectiveness of research drugs, and the accuracy of IHC methods.

### Tumor Mutational Burden

Tumor Mutational Burden (TMB) is defined as the density of nonsynonymous mutations in the coding region of the tumor genome and calculated as mutations per DNA Megabase (Mb) 40. In NSCLC patients with high TMB, the subgroup treated with nivolumab plus ipilimumab had higher 1-year PFS rate (42.6% vs 13.2%), median FPS (7.2 months vs 5.5 months) and better ORR (45.3% vs 26.9%) than those treated with chemotherapy, where TMB cut-off value was 10 mutations per Mb 41. Higher TMB increased the likelihood of neoantigen production, promoting immune recognition and tumor cell killing 42. A statistic correlation between TMB and response rate to anti-PD-1 therapy was observed across different types of tumor 43,44. However, TMB cut-off value varies remarkably across tumor types because of different histological distribution of TMB, it is unrealistic to predict clinical benefits by a universal TMB cut-off value 44. PD-L1 expression and TMB are not related, but increased and higher PD-L1 levels predicts better clinical benefits, according to a study of 240 advanced NSCLC patients treated with PD-1 blockades 45.

Whole exome sequencing (WES) is the gold standard for TMB assessment, but it is only accessible to a few patients due to high cost and complex testing process. Therefore, next-generation sequencing (NGS) for blood TMB (bTMB) is growing popular these days. Rizvi et al. 46 found that in metastatic NSCLC patients with higher bTMB (bTMB ≥20 mutations per Mb), the subgroup treated with durvalumab plus tremelimumab had a significantly better median OS (21.9 months vs 10.0 months) than those treated with chemotherapy. No correlation was found between PD-L1 expression and bTMB 46. Gandara et al. 47 observed that higher bTMB (cut-off value: 16 mutations per Mb) was correlated with remarkable longer OS (13.0 months with atezolizumab vs 7.4 months with docetaxel) in NSCLC patients. In general, good consistency between tissue TMB and blood TMB has been observed, suggesting bTMB as a predictive biomarker for efficacy of ICIs, but more data and evidence are needed 48.

### Mismatch repair deficiency (MMRd)

Mismatch repair (MMR) system takes part in modifying mismatch, insertion and deletion of DNA replication. Le et al. 49 found that metastatic carcinoma patients with MMRd showed significantly higher ORR (40% vs 0%) and PFS rate (78% vs 11%) than those with mismatch repair-proficient, both groups treated with pembrolizumab. Higher ORR and better clinical benefits were observed in patients with ICIs and MMRd across 12 tumor types 50. In the Phase II KEYNOTE-158 Study, Pembrolizumab showed clinical benefits in MMRd/MSI-H (high microsatellite instability) patients with 27 advanced cancers, and outcomes were promising: ORR was 34.3%, the median PFS was 4.1 months and the median OS was 23.5 months 51.

Mutations resulting from MMRd are most frequently located in monomorphic microsatellites, which is called high microsatellite instability (MSI-H). The MMR system usually counts on four vital genes: mutL homologue 1 (MLH1), post meiotic segregation increased 2 (PMS2), mutS homologue 2 (MSH2), and mutS 6 (MSH6), which are detected by related proteins through IHC. If two or more genes altered or inactivated, the tumor is called MSI-H. If one genetic abnormality was observed, it is usually called low microsatellite instability (MSI-L). Or it is called microsatellite stability (MSS) 52. Great consistency (>90%) between MMRd and MSI-H has been observed across cancer types 53. Furthermore, MSH2 expression was found to be associated with high TMB, increased PD-L1 expression and CD8^+^ T cells infiltration in lung adenocarcinoma (LUAD) patients with ICIs, suggesting MSH2 as a potential predictive biomarker 54.

The positive rate of PD-L1 expression was much higher (38.9% vs 16.5%) in solid tumors with MLH1/MSH2 loss than overall samples 55. The significant correlation between positive PD-L1 and MMRd was also described in endometrial carcinomas and colorectal cancer 56,57. Howitt et al. 58 reported that MSI-H was relevant to high TMB and TILs density. It is generally believed that high TMB are associated with MSI-H and MMRd, which is correlated with high level of PD-L1 expression.

## Tumorous factors

### PTEN inactivation

Phosphatase and tension homology deleted on chromosome 10 (PTEN) is a vital tumor suppressor gene controlling the activation of the PI3K-AKT pathway, which participates in the process of cell proliferation. PTEN inactivation is believed to correlate with DNA repair and plays a role in MMRd/MSI-H 59. Loss of PTEN inhibits T-cell mediated tumor killing and lowers the density of TILs in tumor microenvironment, resulting in poor efficacy of immunotherapy 60. Zhao et al. 61 found that PTEN inactivation was enriched in non-responders with glioblastomas, compared with patients who responded to ICIs. Barroso-Sousa et al. 62 demonstrated that PTEN mutations were significantly associated with lower ORR (6% vs 48%), shorter PFS (2.3 months vs 6.1 months) and OS (9.7 months vs 20.5 months) in triple-negative breast cancer patients treated with anti PD-1/PD-L1 therapy. George et al. 63 found that loss of PTEN was correlated with reduced tumor neoantigens which activated T cells *in vitro*, suggesting PTEN inactivation as a predictive biomarker for immunotherapy.

### POLE Mutations

The DNA polymerase epsilon catalytic subunit A (POLE) participates in DNA replication and DNA repair pathways, playing a crucial role in tumor mutations. The loss of POLE raises the tumor mutation rate, resulting in a significant increase of TMB and tumor growth 64. Though uncommon, it was reported that POLE mutations were associated with higher TMB, PD-L1 expression and CD8^+^ TILs density in NSCLC patients treated with immunotherapy, compared with patients with wild-type POLE 65. Increased neoantigen loads and high density of TILs were also observed in patients with POLE mutation across multiple tumor types, suggesting better response to immunotherapy 66,67. Wang et al. 66 reported that the POLE mutation rate was 2.79% across 47721 patients with various cancers, and the mutation rate in non-melanoma skin cancer patients was the highest (16.59%). Under immunotherapy, POLE mutations were correlated with increased OS (34 months vs 18 months with POLE wild-type) 66. In conclusion, current data and studies suggest that POLE mutations predict better clinical benefits for PD-1 blockade therapy, but larger scale of studies are needed for validation.

### Co-Mutation of KRAS and STK11

The correlation between KRAS and STK11 mutations was found based on a large analysis of NGS of 1343 NSCLC tumor samples 68. Skoulidis et al. 69 demonstrated KRAS and STK11/LKB1 co-mutation was relevant to PD-L1 negativity in intermediate or high TMB lung adenocarcinoma (LUAC). Furthermore, patients treated with nivolumab with KRAS and STK11 co-mutations showed a much lower ORR (7.4%), compared with those with KRAS and p53* co-mutations* (35.7%) and KRAS mutations alone (28.6%) 69. In patients with NSCLC, alteration of KRAS/STK11 was found to have influence on tumor microenvironment and decrease PD-L1 expression levels, suggesting a lower response rate to ICIs and poorer prognosis 70,71. Deficiency of STK11/LKB1 was correlated with T cell exhaustion and accumulation of neutrophils with T-cell suppressive effects 71. These studies indicate that KRAS and STK11 mutations predict poorer clinical outcomes for immunotherapy.

### EGFR mutation

EGFR signaling modulates the immunoregulatory effects of MHC I/II and PD-L1 expression on activity of tumor cells and T cells. EGFR mutation (mEGFR) is associated with decreased PD-L1 expression level, lower TMB and decreased density of CD8^+^ T cells infiltration, but the detection of mEGFR is mainly used in NSCLC 72. In advanced NSCLC patients with EGFR mutations (including sensitizing and non-sensitizing mutations) and positive PD-L1 expression (>1%), only 1 in 11 patients (9%) responded to pembrolizumab 73. EGFR sensitizing mutations are the most vital driving gene mutations in lung cancer, and exon19 deletion or L858R point mutation are the most common. Sensitizing mutations tend to induce tyrosine kinase inhibitors resistance and co-mutations, which predict a poor prognosis 74. Oxnard et al. 75 reported that combination of durvalumab and osimertinib (a third generation of EGFR tyrosine kinase inhibitor) would increase the morbidity of interstitial lung disease (38%) in NSCLC patients with mEGFR (L858R or exon19 insertion/deletion), while significant clinical benefits were not observed. However, not all patients with mEGFR could not have clinical benefits from the immunotherapy. Based on the results of the IMpower150 trial, the atezolizumab, bevacizumab, carboplatin, and paclitaxel (ABCP) subgroup had significantly longer OS and PFS than the bevacizumab, carboplatin, and paclitaxel (BCP) subgroup in patients with NSCLC who harbored EGFR sensitizing mutations 76. The clinical efficacy of PD-1/PD-L1 inhibitors combined with tyrosine kinase inhibitors (TKI) was limited in mEGFR patients, but immunotherapy combined with chemotherapy or anti-vascular therapy suggested favorable outcomes. In cancer types of stomach, brain, breast, endometrium and colon, increased EGFR expression levels were also detected, suggesting possibility of EGFR mutations and poor prognosis 77,78. However, studies on EGFR mutations as predictors of immunotherapy have been widely carried out in NSCLC. EGFR-mutated tumors have significantly higher richness and lower T-cell clonality than EGFR-wild-type tumors, although no difference is noted in T cell repertoire, which could be a possible explanation of the lower activity of ICIs.

### TET

DNA hydroxymethylated enzyme Ten-Eleven-Translocation (TET) is capable of demethylation of 5-methylcytosine (5mC) and plays a crucial role in genomic epigenetic regulation. The TET family contains TET1, TET2 and TET3, where mutations of TET2 are thought to be related with hematopoietic malignancy 79. Xu et al. 80 showed that TET2 controlled chemokine and PD-L1 expression, and lymphocyte infiltration through IFNγ-JAK-STAT signaling pathway. Lower activity of TET was associated with decreased TILs and tumor progression and immune evasion. High activity of TET suggested favorable outcomes in patients treated with immunotherapy, which could be enhanced by vitamin C or ascorbate 80. Moreover, Chen et al. 81 found de-ubiquitination enzyme 15 (USP15) could suppress tumor immunity and promote tumor growth through inactivation of TET2. Wu et al. 82 found that TET1-mutant was more common in patients treated with ICIs, and patients with TET1-mutant showed significantly higher ORR (60.9% vs 28.8%) and durable clinical benefits (71.4% vs 31.6%) than patients with TET1-wildtype.

## Biomarkers in peripheral blood

### Neutrophil-to-lymphocyte ratio

Peripheral blood cells are associated with prognosis in patients receiving immunotherapy, especially lymphocyte counts and neutrophil-to-lymphocyte ratio (NLR). Diem et al. 83 found that higher NLR was associated with lower ORR, shorter OS and PFS in NSCLC patients treated with nivolumab. Ren et al. 84 reported that in the advanced NSCLC patients treated with immunotherapy, in the group with TMB>10 mutations per Mb, an association between lower NLR (NLR<2.5) and longer OS and PFS was observed, compared with group with TMB<10 mutation per Mb. Moreover, low absolute lymphocyte count (ALC) was considered to be a biomarker for poorer prognosis. Decreased clinical benefits from immunotherapy and shorter median PFS (60 days vs 141 days) have been observed in patients with lower ALC (ALC < 600 cells/μl) 85. Absolute neutrophil counts (ANC) alone was also relevant to the efficacy of anti PD-1 therapy. Patients with high baseline of ANC (ANC>7500) and elevated NLR (NLR>3) had very low 1- and 2- year survival rates, only 2% and 0% respectively, compared with 43% and 24% for those with lower ANC and NLR 86.

Combined with other established biomarkers, NLR also has the potential for prognosis prediction of patients receiving ICIs. In advanced non-small cell lung cancer (aNSCLC), patients with NLR>5, combined with high PD-L1 expression levels (>80%), showed favorable outcome following first-line pembrolizumab monotherapy 87. But no association between PD-L1 expression and NLR was found 88. Patients with a lower NLR (NLR<2.5) combined with a high TMB (TMB>10) showed favorable OS and PFS than those with a higher TMB and NLR (NLR>2.5). Furthermore, Platelet-to-Lymphocyte ratio (PLR) level below 200 was correlated with longer OS and FPS, which might also have the potential to serve as a potential biomarker for prognosis 89.

### Lactate dehydrogenase

High level of lactate dehydrogenase (LDH) is considered to be a marker for poorer prognosis in various tumors. Kelderman et al. 90 observed that the efficacy of ipilimumab treatment was limited in metastatic melanoma patients with baseline serum LDH greater than twice the upper limit of normal (ULM). Mezquita et al. 91 demonstrated that LDH greater than ULM, and derived neutrophils/(leukocytes minus neutrophils) ratio (dNLR) greater than 3, were considered to be markers for poor treatment outcomes of immunotherapy. However, the level of LDH elevation lacks specificity and may be caused by liver or muscle lesions, which should be excluded from clinical studies.

### Peripheral immune cells

Peripheral immune cells reflect the functions and subtypes of TILs. Gros et al. 92 found that neoantigen-specific CD8^+^ T cells, which could recognize autologous tumors, existed in the circulating PD-1^+^ CD8^+^T-cell population. This study suggested that peripheral blood PD-1^+^CD8^+^ T cells might reflect the change of neoantigen-specific CD8^+^ T cells and predict the efficacy of immunotherapy. Kamphorst et al. 93 reported that 70% of patients with disease progression showed a delayed or absent circulating PD-1^+^CD8^+^ T cells response, while 80% of patients with clinical benefits had a PD-1^+^CD8^+^ T cells response in four weeks. The proliferation of circulating Ki-67^+^PD-1^+^CD8^+^ T cells was also observed in the first or second treatment cycle, associated with a promising treatment efficacy 93. Krieg et al. 94 demonstrated that high frequency of peripheral CD14^+^ CD16^-^ HLA-DR^hi^ monocytes was correlated with increased ORR to anti-PD-1/PD-L1 therapy, and decreased number of peripheral T cells was also observed in responding patients. Higher abundance of CD45RO^+^ T memory cells was observed in responders, suggesting favorable outcomes of immunotherapy 94,95.

### Circulating tumor DNA

Circulating tumor DNA (ctDNA) is generally believed to reflect the mutation load of tumor tissues, and it is more accessible than TMB examinations. Bratman et al. 96 reported that ctDNA baseline level was a sensitive predictor for the efficacy and prognosis in solid tumor patients treated with pembrolizumab, and patients with ctDNA below median baseline levels had a longer OS. The dynamic changes of ctDNA can further predict the clinical benefits. In a cohort study of 73 patients, the ctDNA levels of 33 patients dropped from their baselines, and 14 (42%) of them had objective remission. While among 40 patients with an elevated ctDNA levels from the baselines, only 1 (2%) reached objective remission. Decreased levels of ctDNA after treatment of immunotherapy predict that the patients will have a longer OS 97,98. Gandara et al. 47 reported a positive correlation between ctDNA, obtained by next- generation sequencing method, and tissue TMB. They retrospectively analyzed the outcomes of randomized phase II study POPLAR and phase III OAK trial, defining ctDNA ≥ 16 mutations per megabase (Mut/Mb) as a cut-off point in NSCLC for predicting atezolizumab benefits. But this cut-off value is controversial and may have trouble in applying to other tumor types 99.

### Soluble PD-L1

Like membrane-binding PD-L1, soluble PD-L1 (sPD-L1) inhibits proliferation and activation of T cells, promoting tumor growth and escape. A positive correlation of soluble PD-1 (sPD-1) and favorable clinical outcomes was observed in advanced pancreatic cancer patients. The group with high sPD-L1 levels (sPD-L1 > 0.012 ng/ml) showed a shorter median OS (9.53 months vs 11.92 months) than patients with low sPD-L1 levels. Furthermore, sPD-1 and sPD-L1 levels are closely correlated 100. High baseline levels of sPD-L1 are also associated with increased possibility of tumor progression in patients with melanoma and NSCLC 101,102. However, sPD-L1 levels were not relevant to PD-L1 expression levels of tumor cells 102. The concentration of sPD-L1 is easier to measure than tumor PD-L1 expression levels, but available clinical data is limited and more studied are needed to further unravel the predictive role of sPD-L1.

Besides sPD-L1, exosome PD-L1 levels are also investigated as a predictive biomarker. Theodoraki et al. 103 found that PD-L1 levels on exosomes, instead of levels of sPD-L1, were associated with tumor progression of head and neck squamous cell carcinomas. Fan et al. 104 reported that higher exosome PD-L1 levels, which were an independent prognostic factor, were correlated with lower OS compared with lower level of exosome PD-L1 group in gastric cancer patients. Chen et al. 105 demonstrated that stimulation with interferon-γ (IFN-γ) increased the amount of exosome PD-L1, thereby inhibiting the functions of CD8^+^ T cells and promoting tumor growth.

### Peripheral blood T-cell receptor (TCR)

TILs are not necessarily tumor-recognizing lymphocytes, and the immune response cannot be judged simply by the numbers of TILs. The detection of TCR can reflect T-cell functions of recognizing tumor cells and predict the efficacy of immunotherapy more accurately 106,107. Scheper et al. 107 found that only about 10% of tumor infiltrating T cells could recognize autologous tumor cells by analyzing intra-tumoral TCR repertoire of CD8^+^ T cells in ovarian and colorectal cancer. Postow et al. 108 found that higher evenness (similarities between the rearrangement frequencies of specific V and J genes) of peripheral TCR was associated with better PFS in 12 patients with metastatic melanoma, but no significant difference in OS was observed. Hogan et al. 109 observed that low baseline level of diversity evenness of the TCR repertoire before treatment was related to better FPS and promising response to anti-PD-1 therapy. Moreover, peripheral blood TCR repertoire was correlated to Immune-related Adverse Events (IrAEs) in early stages of treatment. Lack of diversity of CD4^+^ and CD8^+^ TCR was observed in patients with severe IrAEs 110.

### Peripheral cytokines

The alteration of peripheral cytokines can reflect the conditions of tumor microenvironment and T cells response 111. TGF-β is considered as a factor of tumor evasion and immune suppression. High baseline levels of peripheral TGF-β (≥200 pg/mL) are associated with poor prognosis in patients with hepatocellular carcinoma who were treated with pembrolizumab 112. TGF-β, originating from peri-tumor fibroblasts, prevents T cells from infiltrating into the tumor parenchyma, which makes tumor-specific T cells more likely to be distributed in the peri-tumor stroma rather than the intratuminal parenchyma 113. TGF-β inhibitors can increase the sensitivity of anti PD-1/PD-L1 therapy in the mouse models of progressive liver metastatic diseases, which may be due to TGF-β inhibition resulting in an effective and long-lasting cytotoxic T-cell response to tumor cells 114.

IFN-γ, mainly derived from TILs, could promote immune activity and inhibit tumor proliferation, but it also regulates up the expression levels of PD-L1 on tumor cells 115. IFN-γ could activate the JAK2-STAT1 pathway, which suppressed the tumor cell proliferation 116. However, IFN-γ also induces the activation of PI3K-AKT pathway, which could increase PD-L1 expression levels. Blockade of PI3K-AKT pathway would maximize the effect of IFN-γ in anti-tumor therapy 117. The results of phase II POPLAR trial showed increased expression of IFN-γ induced by T-cell effector was correlated to favorable OS in patients with NSCLC who received atezolizumab 118. Furthermore, IFN-γ positive mRNA signature suggested clinical benefits with anti-PD-1/PD-L1 treatment 119. But more clinical data are needed to support this conclusion.

IL-6 is relevant to poor prognosis in patients with NSCLC 120. High levels of IL-6 is associated with significant increase of Treg cells and high expression level of PD-1 on CD4^+^ and CD8^+^ T cells 121. Keegan et al. 122 observed that decreased levels of IL-6 were associated with better FPS in patients with NSCLC who received immunotherapy than those with high or normal levels of IL-6 (median PFS: 11 vs 4 months). These evidences indicate that IL-6 levels might be a potential biomarker for prognosis.

IL-10 is considered as an immunoregulatory cytokine, but it also plays a role in proliferation of CD8^+^ T cells. Li et al. 123 found that IL-10 improved the expression of IFN-γ and inhibited the expression of PD-1 on CD8^+^ T cells in peripheral blood and tumor tissues. Giunta et al. 124 reported higher IFN-γ/IL-10 ratio was observed in responding patients treated with immunotherapy. Boutsikou et al. 125 observed that increased levels of cytokines (TNF-α, IL-1β, IL-2, IL-4 and IL-8) were related to favorable outcomes of anti PD-1/anti PD-L1 therapy, and these cytokines were independent from PD-L1 expression. Additionally, Sanmamed et al. 126 reported a strong correlation between decreased levels of IL-8 and better response to immunotherapy and longer OS in NSCLC and melanoma patients.

**Figure 2:**
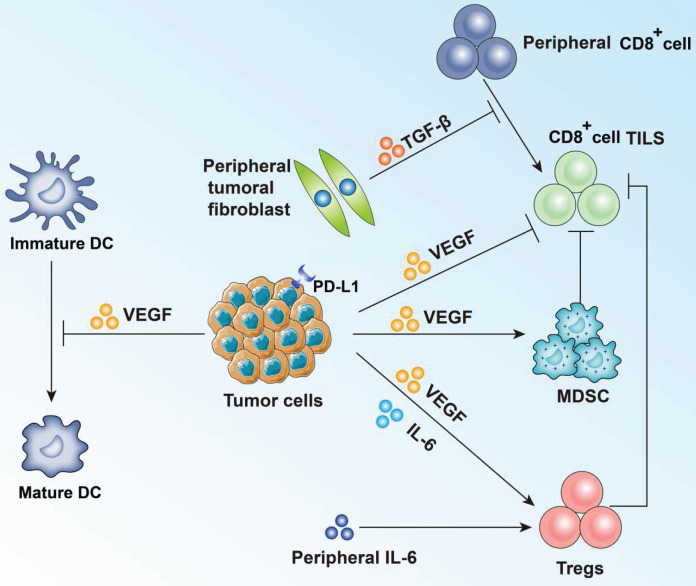
** Summary of effect of VEGF and other main cytokines on tumor cells and TILs.** VEGF, mainly secreted by tumor cells, shows immunosuppressive effect by activating Treg cells and bone marrow-derived suppressor cells (MDSC). VEGF can also inhibit T-cell function directly. Besides, maturation of dendritic cells (DC) is blocked. IL-6 and TGF-β also participate in the immune response and tumor evasion.

Vascular endothelial growth factor (VEGF) plays a crucial role in angiogenesis, which results in tumor growth and metastasis 127. VEGF can inhibit the function of T cells and increase the recruitment of myeleloids-derived suppressor cells (MDSCs) and regulatory T cells (Tregs), and the differentiation and activation of dendritic cells (DCs) are obstructed 128. Wallin et al. 129 demonstrated that anti PD-1 and anti VEGF combined would lead to proliferation of CD8^+^ T cells in tumor microenvironment and improving tumor-specific T-cell migration. Atkins et al. 130 claimed that pembrolizumab combined VEGF inhibitors showed better clinical benefits and appeared to be tolerable in advanced renal cell carcinoma patients. Shibaki et al. 131 observed that high levels of peripheral VEGF were related to worse outcomes and poorer efficacy of immunotherapy in NSCLC patients older than 75 years (Figure 2).

## Immune-related factors

### Beta-2-microglobulin

Beta-2-microglobulin (B2M) is a vital component of human leukocyte antigen class I (HLA-I) molecules, whose mutation inhibits antigen presentation and tumor evasion. Sade-Feldman et al. 132 found that 5 of 17 patients with metastatic melanoma and immunotherapy had point mutations, deletions or loss of heterozygosity (LOH) in B2M. Non-responders had three times more B2M LOH (30% vs 10%) than responders and were associated with poorer OS. Gettinger et al. 133 found that deletion and decreased expression of B2M on tumor cells were correlated with resistance to PD-1 or PD-L1 inhibitors. Class I HLA disruption mediated by B2M deletion leads to ICIs escape in lung cancer. Immunocompetent lung cancer mouse, whose B2M genes were knocked out, also showed the resistance to ICIs. Janikovits et al. 134 found that increased density of PD-1^+^ T cells was associated to B2M mutations, suggesting that deficiency of HLA-I induced by B2M mutations mainly occurred in PD-1^+^ T-cell infiltrating microenvironment.

Pereira et al. 135 reported that 5% lung cancer patients carried B2M mutations, and most of them impair the correct formation of HLA-I complex. Other genetic mutations involved in HLA-I complex maturation had also been observed. The levels of B2M and HLA-I proteins were associated with lower cytotoxic CD8+ lymphocyte infiltration and PD-L1 expression. Chowell et al. 136 found that maximal heterozygosity (different alleles at HLA-I locus) at HLA-I was related to better OS with immunotherapy treatment, compared to those patients who were homozygous for at least one HLA locus. In advanced melanoma patients, the group with the most (>50% cells) and complete loss of major histocompatibility complex(MHC) I expression on tumor cells showed primary resistance to anti cytotoxic T lymphocyte antigen 4 (CTLA-4) therapy, while MHC II expression positive (>1%) on tumor cells suggested favorable response to anti PD-1 therapy 137.

### B7-H4

B7-H4, which belongs to the B7 immunoglobulin superfamily, inhibits tumor immune response by inhibiting T-cell proliferation and cytokines production. And B7-H4 is highly expressed in tumor tissues, but low or no expression in normal tissues 138. B7-H4 also participates in tumor growth and immune escape through suppressing functions and proliferation of T cells 139. Genova et al. 140 found that B7-H4 expression was correlated with the decrease of PFS (1.7 vs 2.0 months) and OS (4.4 vs 9.8 months) in NSCLC patients treated with nivolumab. And no significant association was observed between B7-H4 expression and outcomes of patients receiving platinum-based chemotherapy. Shrestha et al. 141 observed that high expression level of B7-H4 suggested poor prognosis in hepatocellular carcinoma patients treated with immunotherapy. And B7-H4 expression was independently associated with worse prognosis.

### TOX

Scott et al. 142 reported high expression of thymocyte selection-associated high mobility group box gene (TOX) in tumor specific T cells and exhausted T cells during chronic viral infection. The loss of TOX in tumor-specific T cells removes the T-cell failure procedure, but these non-exhausted T cells remains dysfunctional 142. Guo L et al. 143 found that increased TOX expression was positively correlated with higher density of TILs, and high expression of TOX indicated favorable prognosis for various tumor types, especially for lung adenocarcinoma (LUAD). However, Kim et al. 144 found that TOX promoted the exhaustion of CD8^+^ T cells in tumor microenvironment by up-regulating intercellular adhesion molecules, and inhibition of TOX expression was associated with increased efficacy of anti PD-1 therapy. Although TOX showed predictive value for immunotherapy effect, TOX measurement was hard to be applied in daily clinical practice.

## Gut microbiota

Gut microbiota could influence host immune response and have an impact on the efficacy of PD-1 based immunotherapy (Table 4). Sivan et al. 145 found that oral administration of *bifidobacteria* alone was associated with enhanced function of dendritic cells (DC), which resulted in increased density of CD8^+^ T cells in tumor microenvironment and better effect of ICIs. But this phenomenon disappeared after fecal transfer. Routy et al. 146 found that oral supplemented or fecal microbiota transplanted *Akkermansia muciniphila* would enrich the CRCX3^+^ CD4^+^ and CD8^+^ T cells in tumor bed, and enhance cytotoxic CD8+ lymphocyte infiltration and T-cell immune functions, which was correlated with improvement of the efficacy of immunotherapy. *Enterococcus hirae* was observed to help increase the ratio of CD8^+^ T-cell/Treg after transferring to secondary lymphoid organs 147. Enrichment of *Barnesiella intestinihominis* in the colon was related to the increase of tumor infiltrating T cells 147. Furthermore, Matson et al. 148 found that* Bifidobacterium longum*, *Colinthia*, and *Enterococcus faecalis* were more abundant in the responders, which could be repeated in germ-free mouse by fecal microbiota transplantation from the responders. Gopalakrishnan et al. 149 observed much higher alpha diversity and relative abundance of bacteria of the *Ruminococcaceae* family in melanoma patients responding to PD-1 based immunotherapy. Therefore, the dominant presence of different species of microbe in different cancers conferred sensitivity to immunotherapy, and analysis of gut microbiota composition can be helpful to predict the efficacy of anti-PD-1/PD-L1 treatment.

## Biomarkers for hyper progressive disease

Hyper progressive disease (HPD) is referred to as abnormal tumor growth related to very poor prognosis (Table 5). No consensus has been reached on the definition of HPD, but it is acknowledged that if the tumor growth kinetics and/or tumor growth rate increased by at least two times during treatment, it can fall into this category 150,151. Ferrar et al. 152 found that HPD was more common in NSCLC patients who received immunotherapy (13.8%, 56 in 406 patients) than those treated with chemotherapy (5.1%, 3 in 59 patients). Kim et al. 153 also observed this phenomenon, and they found that low frequency of CD8^+^ T cells, CCR7^-^CD45RA^-^ T memory cells and high frequency of exhausted T cells in peripheral blood were associated with higher occurrence of HPD and worse outcome in NSCLC patients treated with ICIs.

Advanced age is a risk factor for HPD. The incidence rate of HPD is significantly higher in patients over the age of 65 than those under 65 (37% vs 19%) 154. Lo Russo et al. 155 found that increased infiltration of M2 tumor-associated macrophages (TAMs), which participated in immunotherapy, was related to HPD. Increased infiltration of M2 TAMs in tumor microenvironment activated Treg cells and accelerated apoptosis of effector T cells to suppress immune response 156. Furthermore, Chen et al. 157 demonstrated that high concentrations of ctDNA and MIKI67 mutations were correlated with HPD in 22 advanced NSCLC patients treated with immunotherapy.

## Conclusion

It is​ generally believed that PD-L1 expression is the main predictive biomarker for anti PD-1 therapy. But PD-L1 expression is not an independent factor, which could be affected by TMB, MMRd and other predictive factors. PD-L1 expression combined with other established biomarkers, such as TMB and TILs, could better predict the immunotherapy effects in certain tumor types. Mutations in tumor genes are considered to be the initiating factors for tumor growth and progression. Some specific mutations, such as POLE and PTEN, are greatly associated with efficacy of ICIs. However, whole exome-sequencing (WES) or next-generation sequencing (NGS) has not been widely applied in clinical practice due to high cost and technical factors. Proliferation and activation of T cells plays a crucial role in immunotherapy. T-cell related factors can reflect immunotherapy effects more intuitively. But these factors have not been universally tested in clinical practice. Peripheral biomarkers are more accessible than established biomarkers mentioned above. However, these factors are more susceptible to interference. Some biomarkers, like high levels of LDH, have low specificity. The examinations of major biomarkers are limited to laboratory studies and there is still a long way to go between laboratory researches and clinical applications.

There are a large number of novel biomarkers emerging correlated with tumor progression or regression in the process of immunotherapy treatment, which are not fully covered in this article. Additionally, some of these factors may have values in prognosis prediction for the patients who are not treated with PD-1 inhibitors. Overall, more data and evidence are needed to support the predictive values of these emerging biomarkers.

## Figures and Tables

**Table 1:** Predictive effect of PD-L1 expression for PD-1/PD-L1 inhibitors

Immunological checkpoint inhibitor	Study	Tumors	Participants	Cut-off	Efficacy (95% CI)	Ref
Pembrolizumab	NCT01295827	Advanced NSCLC	n=101	<1% 1%-49% ≥50%	Median PFS, months 3.5 (2.1 to 19.0) Median PFS, months 4.2 (3.1 to 6.4) Median PFS, months 12.5 (6.2 to NR)	33
	NCT01295827	Melanoma	n=451	<10% ≥10%	Median PFS, months 5.6 (4.4 to 8.1) Median PFS, months 2.8 (2.7 to 2.8)	34
	NCT02335424	Urothelial carcinoma	n=370	All patients ≥10%	ORR: 24% (20% to 29%) ORR: 38% (29% to 48%)	6
	NCT02255097	Squamous cell carcinoma	n=171	<50% ≥50%	ORR: 13% (7% to 20%) ORR: 27% (15% to 42%)	35
Nivolumab	NCT01721772	Metastatic melanoma	n=418	<5% ≥5%	ORR 33.1% (25.2% to 41.7%) ORR 52.7% (40.8% to 64.3%)	36
	NCT02387996	Urothelial carcinoma	n=265	<1% ≥1%	ORR: 16.1% (10.5% to 23.1%) ORR: 23.8% (16.5% to 32.3%)	37
Nivolumab & Ipilimumab	NCT02477826	Acute myeloid leukemia	n=70	<1% ≥1%	Median OS, months: 17.2 (12.8 to 22.0) Median OS, months: 17.1 (15.8 to 20.1)	38
Atezolizumab	NCT02008227	NSCLC	n=425	<1% ≥1%	Median PFS, months 12.6 (9.6 to 15.2) Median PFS, months 20.5 (17.5 to NR)	8
	NCT02108652	Urothelial carcinoma	n=119	<5% ≥5%	ORR: 21% (11% to 35%) ORR: 28% (14% to 47%)	39

**Table 2:** Tumor microenvironment immune types (TMIT)

Classification	TIL and PD-L1 expression	Characteristics
TMIT 1	TIL^+^ PD-L1^+^	Sensitive to immunotherapy
TMIT 2	TIL^-^ PD-L1^-^	Low response rate to ICIs
TMIT 3	TIL^-^ PD-L1^+^	Dysfunction of T cells
TMIT 4	TIL^+^ PD-L1^-^	Lack of target

**Table 3:** Predictive effect of some peripheral immune cells

Predictive factors	Cancer types	Predictive effect	Ref
Ki-67^+^ PD-1^+^ CD8^+^ T cells	NSCLC	Favorable objective response	93
CD14^+^CD16^-^HLA-DR^hi^ monocytes	Stage IV melanoma	Favorable objective response	94
CD45RO^+^ T memory cells	NSCLC	Favorable objective response	25
FOXP3^+^ Treg cells	metastatic gastric cancer	Poor objective response	26

**Table 4:** Favorable predictive effect of gut microbiota

Favorable predictive factors	Cancer types	Model	Ref
*Bifidobacterium*	Melanoma	Mouse	145
*Faecalibacterium*	Metastatic melanoma	Human	158
*Akkermansia muciniphila*	Metastatic melanoma	Mouse/human	149
*Bacteroides*	Melanoma	Human/mouse	159
Higher diversity of gut microbiota	Metastatic melanoma	Mouse/human	149

**Table 5:** Predictive biomarkers for HPD

Biomarkers	Cancer types	Predictive effect on HPD	Ref
MDM2 family amplification	Not mentioned	Positive (4 in 6 patients, 67%)	160
EGFR mutation	Not mentioned	Positive (2 in 10 patients, 20%)	160
Elevated NLR	NSCLC	Positive	153
Elevated levels of LDH, ANC and CRP	Advanced gastric cancer	Positive	161
Ki67^+^ PD1^+^ Treg cells	Gastric cancer	Positive	162
CCR7^-^ CD45RA^-^CD8^+^T memory cells	NSCLC	Negative	153
TIGIT^+^PD1^+^CD8^+^ T cells	NSCLC	Positive	153

## Abbreviations

PD-1: programmed cell death protein 1
PD-L1: programmed cell death ligand 1
TMB: tumor mutation burden
ICIs: immune checkpoint inhibitors
NSCLC: non-small cell lung cancer
TILs: Tumor infiltrating lymphocytes
TCRs: T-cell antigen receptors
TMIT: tumor microenvironment immune types
Mb: Megabase
MMRd: Mismatch repair deficiency
MSI-H: high microsatellite instability
MSI-L: low microsatellite instability
MLH-1: mutL homologue 1
PMS2: post meiotic segregation increased 2
MSH2: mutS homologue 2
MSH6: mutS 6
MSS: microsatellite stability
LUAD: lung adenocarcinoma
EGFR: epidermal growth factor receptor
TKI: tyrosine kinase inhibitors
TET: hydroxymethylated enzyme Ten-Eleven-Translocation
5mC: demethylation of 5-methylcytosine
USP15: de-ubiquitination enzyme 15
NLR: neutrophil-to-lymphocyte ratio
ALC: absolute lymphocyte count
ANC: absolute neutrophil counts
PLR: platelet-to-lymphocyte ratio
LDH: lactate dehydrogenase
ULM: upper limit of normal
dNLR: derived neutrophils/(leukocytes minus neutrophils) ratio
ctDNA: circulating tumor DNA
sPD-L1: soluble PD-L1
IFNγ: interferon-γ
IrAEs: Immune-related Adverse Events
TGF-β: transforming growth factor-β
VEGF: vascular endothelial growth factor
IL-6: interleukin- 6
IL-8: interleukin- 8
IL-10: interleukin- 10
MDSCs: myeleloids-derived suppressor cells
Tregs: regulatory T cells
DCs: dendritic cells
B2M: beta-2-microglobulin
HLA-I: human leukocyte antigen class I
LOH: loss of heterozygosity
MHC: major histocompatibility complex
CTLA-4: cytotoxic T lymphocyte antigen 4
TOX: thymocyte selection-associated high mobility group box gene
TAMs: tumor-associated macrophages
CRP: C-reactive protein
IHC: immunohistochemical
PET: positron emission tomography
WES: Whole exome sequencing
NGS: next-generation sequencing
bTMB: blood tumor mutation burden
PFS: progression-free survival
OS: overall survival
ORR: objective response rate
HPD: hyper progressive disease
